# The Tongue in Influenza

**Published:** 1899-09

**Authors:** 


### The Tongue in Influenza.

M. d'Hotel calls attention to a character of the tongue constituting a pathognomonic sign of that mysterious affection known as influenza. If the malady is observed during the first few hours of its invasion, the tongue may not present any abnormal feature, but the following day it is invariably covered with a white coating more or less thick towards the center. Later on, according as the affection is of a benign type, or becomes complicated or prolonged, the lingual coating is seen to diminish from the point, or, on the contrary, to remain. The *label* of influenza is in general the last sign to disappear, and it is not rare to observe three weeks after the *debut*, a remainder of a whitish triangle at the base of the tongue, indicating that the patient not only has been through the malady, but also that he is not yet absolutely free from the morbid condition, although the general state of his functions may appear regular; an imprudence on his part, a cold, might provoke broncho-pneumonia, gastro-enteritis or some other complication. Another characteristic of this lingual deposit is to redden litmus paper when rubbed on it, and not only during the first days of the malady, but as long as there remains a trace of it on the tongue.

This acidity persists as long as there remains any trace of the fur, and is a natural indication of the treatment, which is that of frequently rinsing the mouth with an alkaline solution such as Vichy water, followed by the internal administration of the same. It is evident that influenza cannot be cured more than typhoid fever, but M. d'Hotel affirms that complications were much rarer where the alkaline treatment was used.—*Paris Cor. Med. Press and Circular.*

